# The Necessity of Lymph Node Dissection Between Sternocleidomastoid and Sternohyoid Muscles in pN1b Papillary Thyroid Carcinoma

**DOI:** 10.3389/fendo.2022.865621

**Published:** 2022-04-25

**Authors:** Yuanpeng Zhai, Litao Ruan

**Affiliations:** Department of Ultrasound Medicine, The First Affiliated Hospital of Xi’an Jiaotong University, Xi’an, China

**Keywords:** papillary thyroid carcinomas, lymph node between sternocleidomastoid and sternohyoid muscle, risk factor, neck dissection, lymph node metastasis

## Abstract

**Background:**

This study aimed to evaluate the association between clinicopathologic variables and metastasis of the lymph node (LN) between the sternocleidomastoid and sternohyoid muscles (LNSS) to clarify the necessity of LNSS dissection in papillary thyroid carcinomas (PTCs).

**Methods:**

A total of 219 patients undergoing unilateral or bilateral neck dissection for PTCs were prospectively enrolled. The associations between clinicopathologic variables and LNSS metastasis were evaluated by univariate and multivariate analyses.

**Results:**

LNSS was present in 108 (40.1%) neck dissection samples and in 76 (34.7%) patients. Positive LNSS occurred in 40/269 (14.9%) neck dissection samples and in 20/219 (9.1%) patients. Univariate analysis showed that tumor stage, number of positive nodes in level III, and number of positive nodes in level IV were related to LNSS metastasis. Multivariate analysis confirmed that T3/4 stage tumors and >2 positive LNs in level IV independently increased the risk of LNSS metastasis.

**Conclusions:**

The low rate of LNSS metastasis would deem routine dissection unnecessary; however, LNSS would require excision if advanced stage tumors or level IV LN metastasis were present.

## Introduction

Papillary thyroid carcinoma (PTC) is the most common endocrine malignancy, and accounts for nearly 90% of differentiated thyroid carcinomas (1). Lymph node (LN) metastasis usually develops in PTCs, with a reported incidence of up to 90% (2–4). Routine central neck LN dissection is required for all PTC patients in China, while lateral neck dissection is performed for pathologically positive lateral LN (5).

Excision of LNs located between the sternocleidomastoid and sternohyoid muscles (LNSS) is often overlooked because of it is located in the suprasternal space and falls outside the neck levels VI and level II–V (6–10). Since Sun et al. (6) first described the metastasis pattern of LNSS, other researchers have continued to emphasize the significance of LNSS dissection (7–10). The rate of LNSS metastasis varies from 7.4% to 22.6%, yet the risk factors for LNSS metastasis remain unknown. This study aimed to evaluate the association between clinicopathologic variables and LNSS metastasis to clarify the necessity of LNSS dissection in PTCs.

## Patients and Methods

### Ethics Consideration

The study was approved by Xi’an Jiaotong University Institutional Research Committee. The participants were asked to sign an informed consent form. All methods were performed in accordance with the relevant guidelines and regulations and adhered to the ethical standards of the institutional and national research committee as well as with the 1964 Helsinki Declaration.

### Patient Selection

From November 2020 to November 2021, 250 consecutive patients with primary cN1b PTC sought medical service in our cancer center. After preoperative fine needle aspiration and frozen section, 219 patients were enrolled for unilateral or bilateral neck dissection. They had no history of any other malignancy or neck surgery and information on their demography, pathology, tumor stage (based on the 8^th^ AJCC classification) and treatment were recorded and analyzed.

### Surgical Treatment

**Figure 1:**
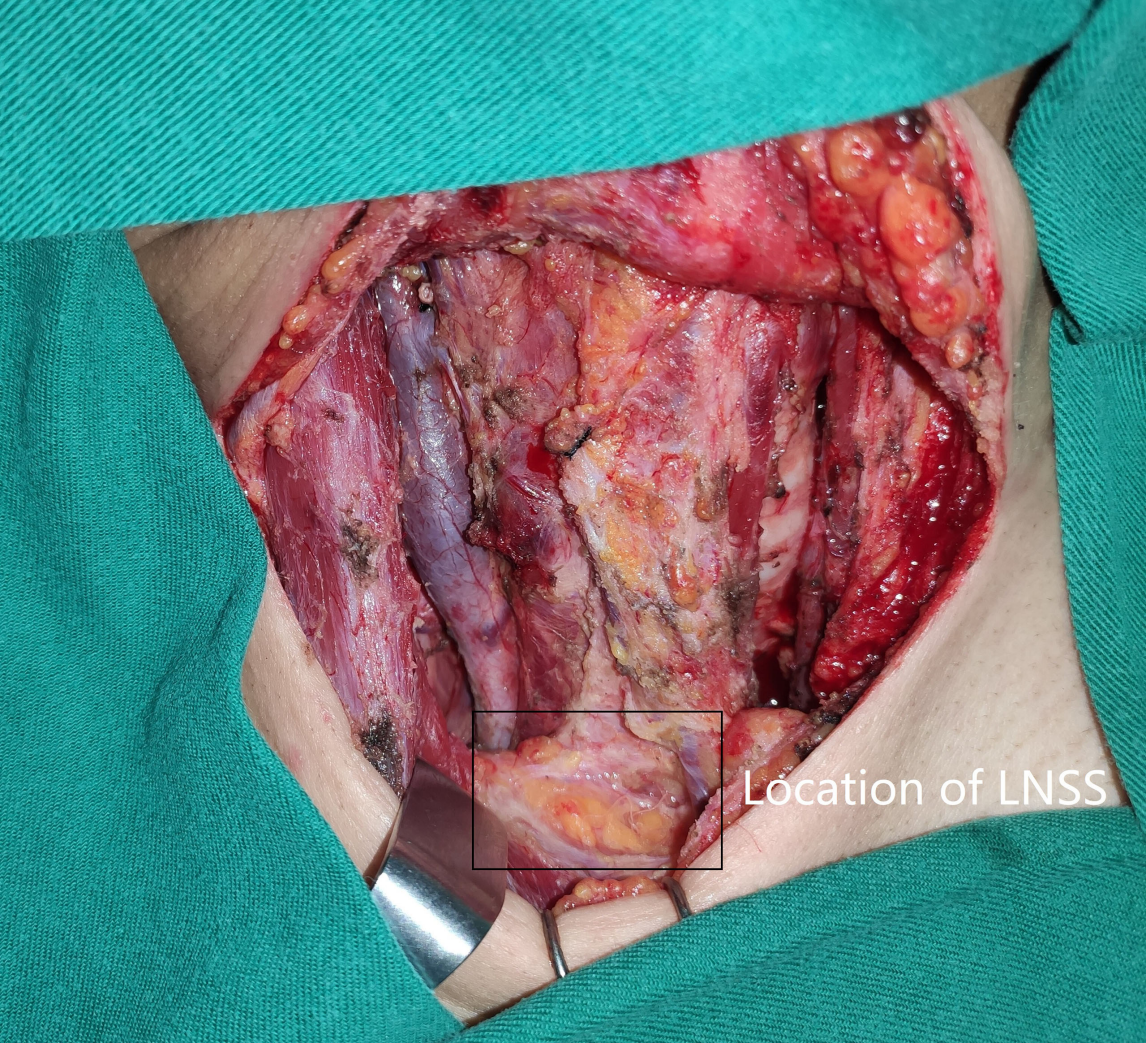
Location (black box) of lymph node between sternocleidomastoid and sternohyoid muscles (LNSS).

**Figure 2:**
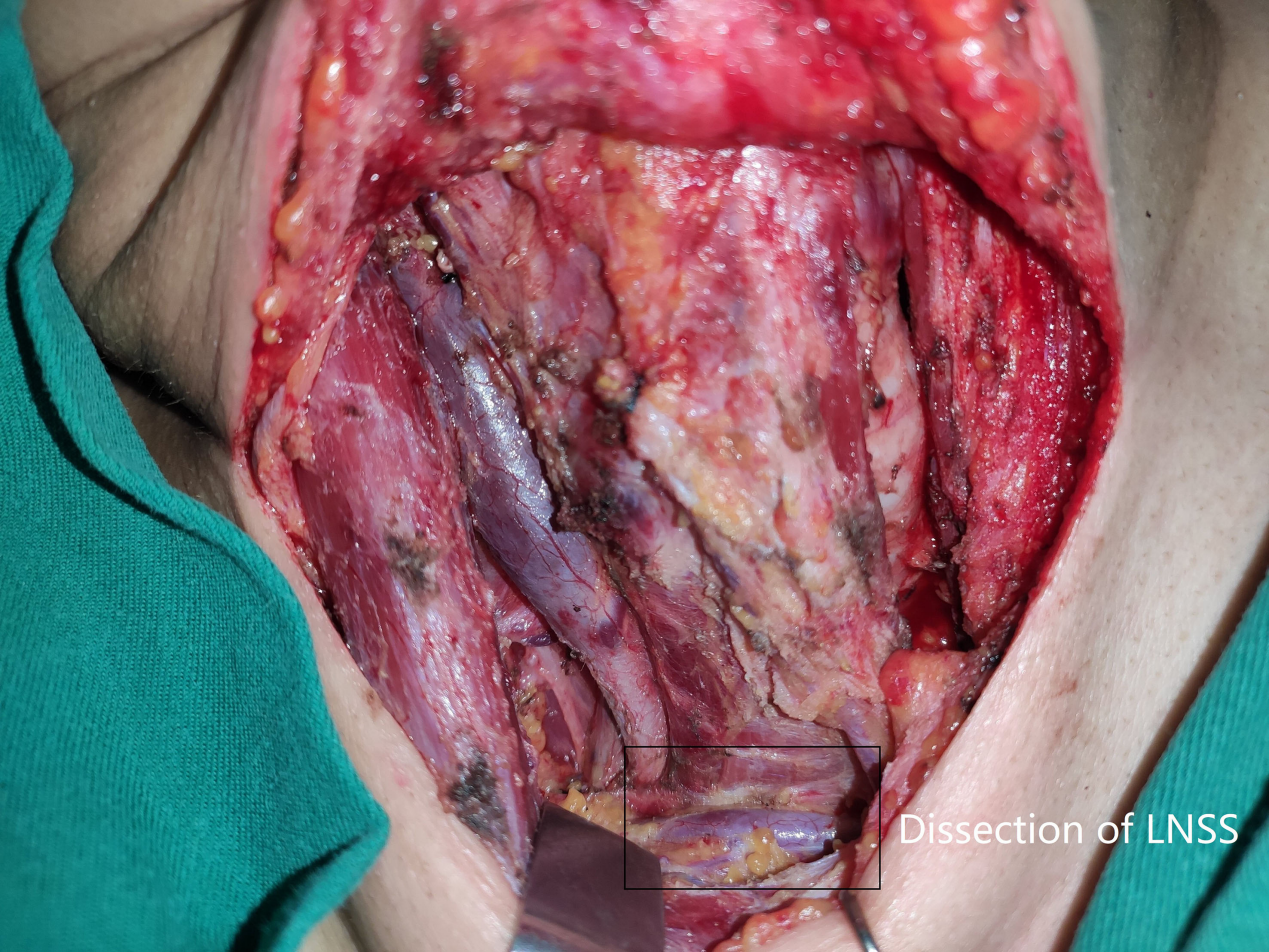
Dissection (black box) of lymph node between sternocleidomastoid and sternohyoid muscles (LNSS).

Neck status was evaluated using palpation, ultrasound, and computed tomography (CT). Suspicious LNs pertaining to level II to V were further assessed by measuring the thyroglobulin levels in the washout fluid of the preoperative fine needle aspiration (11) or by intraoperative frozen section. If positive lateral LN were confirmed, the lymphoid adipose tissue between sternocleidomastoid and sternohyoid muscles and LNs of level II to IV/V were dissected, and every subgroup was separately sent for pathological analysis. LNSS was defined as follows (**Figures 1**, **2**): superiorly by the intersection of the sternocleidomastoid and sternohyoid muscles, inferiorly by the suprasternal fossa and clavicle, anteriorly by the sternocleidomastoid muscle, posteriorly by the sternohyoid muscle, internally by the border of the sternocleidomastoid, and laterally by the border of the sternohyoid muscle (6, 7).

### Statistical Analysis

The association between clinicopathologic variables and LNSS metastasis was evaluated by the chi-square test, and the variables which indicated significance in univariate analyses were then analyzed in a multivariate binary logistic regression model to determine the independent predictors. All statistical analyses were performed using SPSS 20.0, and p<0.05 was considered to be significant.

## Results

### Baseline Data

The 219 patients comprised 62 men and 157 women. The median age was 53 (range: 18-76) years. Tumor stages were distributed as T1 in 34 patients, T2 in 45 patients, T3 in 88 patients, and T4 in 52 patients. Multifocality occurred in 117 patients. Comorbid Hashimoto’s thyroiditis developed in 86 patients and mutation of BRAF V600E gene occurred in 163 patients. All patients underwent total thyroidectomy, of which 50 patients received additional bilateral neck dissection and 169 patients received additional unilateral neck dissection. The mean ± SD of resected LNs for each neck dissection sample was 28 ± 6.4. The mean of positive LNs in levels II, III, IV, and VI were 1.0 ± 0.7, 2.2 ± 1.3, 2.5 ± 1.6, and 2.8 ± 1.5, respectively.

### LNSS

LNSS were present in 108 neck dissection samples (40.1%) and in 76 patients (34.7%). The mean LN size was 4.0 ± 2.8 mm, while the median of the incidence of LNSS was 1 with an interquartile range of 1-5. Positive LNSS was identified in 40/269 (14.9%) neck dissection samples and in 20/219 (9.1%) patients. The mean size of the positive LNSS was 4.5 ± 2.4 mm.

### Association Between LNSS Metastasis and Clinicopathologic Variables

In the 130 patients with T3/4 tumors, the rate of LNSS metastasis (11.4%) was significantly higher than in the 89 patients with T1/2 tumors (4.5%) (*p*=0.049). When the 219 patients were divided into three groups based on the number of positive LNs in level III, a significant difference (*p=*0.048) was noted in the rates of LNSS metastasis in patients with 0, 1-2, and >2 positive LNs (4.4%, 9.9%, and 17.0%, respectively). Division of the 219 patients into three groups based on the number of positive LNs in level IV also yielded a significant result (*p*=0.016) when the rates of LNSS metastasis in patients with 0, 1-2, >2 positive LNs (2.6%, 9.9%, and 16.7%, respectively) were compared. There were no significant relationships between LNSS metastasis and other variables (**Table 1**).

**Table 1:** Relationship between lymph node between sternocleidomastoid and sternohyoid muscle (LNSS) metastasis and clinicopathologic features.

Variable	LNSS metastasis		p
	Yes (n=20)	No (n=199)	
Age			
<55	11	103	
≥55	9	96	0.782
Sex			
Male	5	57	
Female	15	142	0.730
Tumor stage			
T1+T2	4	85	
T3+T4	16	114	0.049
BRAF V600E mutation			
No	3	53	
Yes	17	146	0.256
Hashimoto's thyroiditis			
No	14	119	
Yes	6	80	0.373
Multifocality			
No	6	96	
Yes	14	103	0.119
Number of positive nodes in level II			
0	13	146	
1-2	5	35	
>2	2	18	0.727
Number of positive nodes in level III			
0	4	87	
1-2	8	73	
>2	8	39	0.048
Number of positive nodes in level IV			
0	2	76	
1-2	8	73	
>2	10	50	0.016
Number of positive nodes in level VI			
0	3	42	
1-3	10	85	
>3	7	72	0.754

In multivariate analysis, the advanced tumor stage and number of positive LNs in level IV metastasis were considered to be the independent predictors for LNSS metastasis. Compared to T1/2 tumors, T3/4 tumors were associated with a 2.4-fold increase in the risk of LNSS metastasis. Compared to tumors without level IV metastasis, tumors with >2 positive LNs in level IV metastasis were associated with an 2.1-fold increase in the risk of LNSS metastasis (**Table 2**).

**Table 2:** Multivariate analysis of the predictors for lymph node between sternocleidomastoid and sternohyoid muscle (LNSS) metastasis.

Variable	p	OR [95%CI]
Tumor stage (T3+T4 vs T1+T2)	0.022	2.456 [1.327-4.665]
Number of positive nodes in level III		
0		
1-2	0.354	2.678 [0.766-5.332]
>2	0.116	3.276 [0.876-8.116]
Level IV metastasis		
0		
1-2	0.111	1.665 [0.887-4.377]
>2	0.014	2.122 [1.227-6.432]

## Discussion

The most important finding in the current study was that although nearly one third of the patients had LNSS, LNSS metastasis was relatively uncommon. It was most likely to occur with the T3/4 tumors or along with level IV metastasis simultaneously. The rate of LNSS metastasis was found to be low and routine dissection was unnecessary. However, the excision of LNSS was required if there was a presence of advanced stage tumors or LN metastasis in level IV.

The suprasternal space, also known as Burns space, is a narrow interval between the superficial and deep layer of the cervical fascia, located above the manubrium of the sternum. It contains the sternal heads of the sternocleidomastoid, the lower portions of the anterior jugular veins and their transverse connecting branch as well as a small quantity of areolar tissue (12). LNSS was anatomically a part of this space (6–10, 13); however, according to the official definition of level III-VI (14), LNSS does not belong to any of these groups. Moreover, owing to the developmental variation, LNSS is not always present. Song et al. (7) noted LNSS in 75.8% of 264 neck dissection samples, while Hao et al. (8), Sun et al. (9), and Zhang et al. (10) reported LNSS in about 70% of their patients. The incidence was apparently lower than reported in our study. Usually, the detection rate of LNs is associated with a number of factors including the pathologist’s ability to identify the LNs, the surgeon’s ability to dissect the LNs, and the level of dissection (15). It was noted that the size of the LNSS in the current study was very small, representative of the eligibility of our surgeons and pathologists. Thus, it is reasonable to conclude that other factors may account for this variation, such as differences in the study design and inclusion criteria. Moreover, we might be the first to depict the small size of LNSS, which is not easily detected in a CT scan, resulting in the frequent neglection of this special group.

There is a paucity of studies on the metastasis of LNSS and to our knowledge, only five published studies have focused on this issue (6–10). Sun et al. (6) were the first to describe positive LNSS in 22.6% of their 115 neck dissection samples from 115 patients. Consistent with our study findings, Song et al. (7) reported a 17.8% rate of LNSS metastasis in 234 PTC patients undergoing 264 neck dissections, while metastatic incidences of 7.4%, 23.5%, and 14.3% were reported by Hao et al. (8), Sun et al. (9), and Zhang et al. (10), respectively. These findings indicated that the rate of LNSS metastasis was relatively low, raising the important question of when LNSS should be resected.

Predictors for LNSS metastasis remain unclear. Based on a study comprising 115 patients, Sun et al. (6) found a significant association between LNSS metastasis and the level III and level IV nodal metastasis, the lateral LN metastasis, and a primary site in the inferior pole. However, the authors did not perform a multivariate analysis to confirm the independence of these factors. In another study consisting of 264 neck dissections from 234 patients, the authors noted that age over 45 years, a tumor located in the inferior lobe of thyroid, LN metastasis at level IIb, and LN metastasis at level III were all independent metastatic risk factors for LNSS (7). The role of level IIb metastasis was not clear. The rate of level IIb metastasis in PTC was usually very low, thus the routine dissection of level IIb was not deemed to be necessary. However, resection of level IIb was indicated if there were positive LNs in more than three levels (16). Additionally, because of the anatomical distance between level IIb and LNSS, it was hard to assess causality. Sun et al. (9) confirmed the independence of the original tumor size and level IV nodal metastasis in increasing the risk of LNSS metastasis. All the findings emphasized the significance of level IV metastasis; however, they failed to identify the risk as per the number of positive LNs. Cervical LNs with metastasis >6 were associated with an elevated risk of LNSS metastasis (8). Moreover, the current study reiterates the fact that the risk of LNSS metastasis was significantly increased when there were more than two positive LNs in level IV.

Tumor stage was another important predictor. Usually, thyroid LNs only drain to deep cervical LNs, but rarely metastasize to superficial cervical LNs (17). However, advanced stage tumors can invade peripheral tissues such as anterior cervical muscles; cancer cells can then cause LNSS enlargement through the superficial cervical lymphatic drainage system. This viewpoint was confirmed by Zhang et al. (10), who reported that 88.9% of the PTCs with strap muscle involvement had LNSS metastasis, a rate higher than the 19.4% observed in PTCs without strap muscle involvement. Additionally, pre-laryngeal LNs are considered to have communicating branches with superficial cervical lymphatic drainage systems (18). However, we failed to identify the effect of level VI metastasis on LNSS metastasis in this study.

The limitations of the current study must be acknowledged: the prognostic value of LNSS was not analyzed because the follow-up data was not included.

In summary, the rate of LNSS metastasis was low, routine dissection was unnecessary. However, the excision of LNSS was required if there was a presence of advanced stage tumors or LN metastasis in level IV.

## Data Availability Statement

The original contributions presented in the study are included in the article/supplementary material. Further inquiries can be directed to the corresponding author.

## Ethics Statement

The study was approved by Xi’an Jiaotong University Institutional Research Committee. The participants were asked to sign an informed consent form. The patients/participants provided their written informed consent to participate in this study.

## Author Contributions

The conception and design of the work: YZ and LR. The collection and analysis of the data: YZ and LR. The interpretation of the statistic results: YZ and LR. Manuscript writing and revision: YZ and LR. All authors contributed to the article and approved the submitted version.

## Conflict of Interest

The authors declare that the research was conducted in the absence of any commercial or financial relationships that could be construed as a potential conflict of interest.

## Publisher’s Note

All claims expressed in this article are solely those of the authors and do not necessarily represent those of their affiliated organizations, or those of the publisher, the editors and the reviewers. Any product that may be evaluated in this article, or claim that may be made by its manufacturer, is not guaranteed or endorsed by the publisher.
